# Olfaction as a Marker for Psychiatric and Neurological Diseases

**DOI:** 10.3390/brainsci12010023

**Published:** 2021-12-25

**Authors:** Wissam El-Hage

**Affiliations:** 1 Department of Psychiatry, CHRU de Tours, 37000 Tours, France; wissam.elhage@univ-tours.fr; 2 Université de Tours, U 1253, iBrain, CIC 1415, Inserm, 37000 Tours, France

Olfaction is one of the oldest senses among the five human senses. It contributes in identifying foods, dangers, objects, animals or other people, thus playing a key social and emotional role. Olfactory signals in humans influence their behavior, interpersonal relationships, their tastes, and their personality. Olfaction can be measured and altered at different levels. Olfactory dysfunction has various forms (e.g., anosmia, partial anosmia, hyposmia, microsmia, dysosmia, phantosmia, olfactory agnosia, and hyperosmia) and has been associated with many medical conditions (e.g., following accidents, psychiatric and neurological conditions, medical conditions, aging, medical interventions, and exposure to environmental chemicals, etc.). Olfactory dysfunctions and biases can be characterized and thus constitute potential discriminatory markers for psychiatric and neurological disorders, which dysfunctions can be considered as either a consequence of the disease or a vulnerability factor. This Special Issue published six contributions about innovative diagnostic and therapeutic approaches of olfactory disorders, and original research articles advancing our understanding of olfaction as a marker for psychiatric and neurological diseases.

The first paper explored olfactory processing in depression, comparing olfactory functions between unipolar and bipolar patients in order to identify potential state/trait markers. “Olfactory Memory in Depression: State and Trait Differences between Bipolar and Unipolar Disorders” by Kazour and colleagues [1] evaluated long-term olfactory memory recognition function in depressed patients (unipolar and bipolar) in comparison to remitted patients and healthy controls. They showed that olfactory recognition memory is altered in depressed patients (both unipolar and bipolar) compared to healthy controls, constituting a state marker of depression. Moreover, this alteration persisted in euthymic remitted bipolar patients, constituting a possible trait marker of bipolarity. This study provided thus strong evidence that sensory olfactory markers may be useful clinical tools in differentiating between unipolar and bipolar depressive episodes.

In the second paper, the authors made the hypothesis of reciprocal relationships between mindfulness, interoception and exteroception (including olfaction and emotion). “Mindfulness, Interoception, and Olfaction: A Network Approach” by Lefranc and colleagues [2] explored how mindfulness interacts with information about the body’s physiological state (interoception) using interaction networks and the state of the external world (exteroception). They demonstrated that interoception awareness is strongly connected with both the mindfulness disposition and the hedonic value of odors. They suggested that olfaction plays a role in the fine-tuned interplay between the brain and the body underlying emotional abilities to respond appropriately in a constantly changing environment.

The following two articles examined the relationship between olfactory function and Alzheimer’s disease. “Functional Connectivity between the Resting-State Olfactory Network and the Hippocampus in Alzheimer’s Disease” by Lu and colleagues [3] investigated the resting-state functional connectivity between the olfactory network and the hippocampus, in subjects who were categorized as age-matched cognitively normal, early mild cognitive impairment, late mild cognitive impairment, and with Alzheimer’s disease. They demonstrated that functional connectivity between the olfactory network and hippocampus may be a sensitive marker for Alzheimer’s disease progression, preceding gray matter volume loss.

“Emotional and Phenomenological Properties of Odor-Evoked Autobiographical Memories in Alzheimer’s Disease” by Glachet and colleagues [4] investigated emotional characteristics (arousal and valence) and subjective reliving of odor-evoked autobiographical memories in Alzheimer’ disease. They evaluated whether odor exposure may enhance the subjective reliving and the emotional properties of autobiographical memories in Alzheimer’ disease. They showed that odor-evoked autobiographical memories are more anchored in a spatiotemporal context than memories retrieved without odor (higher arousal, subjective reliving, and more positive memories). They also found that odor-evoked emotion and subjective reliving were associated with depressive symptoms in Alzheimer’ disease. Thus, odor may be a useful cue to trigger more detailed, vivid, and positive events in Alzheimer’ disease.

The fifth paper explored the link between dystonia and diminished sense of smell. “Olfaction as a Marker for Dystonia: Background, Current State and Directions” by Herr and colleagues [5] reviewed evidence on dystonia and its pathophysiology, including the key neural structures involved. Furthermore, they discussed involvement of these brain structures in the chemical senses to provide an overview on how olfactory deficits may occur in dystonia.

Finally, “Chemosensory Event-Related Potentials and Power Spectrum Could Be a Possible Biomarker in 3M Syndrome Infants?” by Invitto and colleagues [6] analyzed the cortical olfactory response through chemosensory event-related potentials and power spectra calculated by electroencephalogram signals recorded in infants with 3M syndrome, a rare autosomal recessive dwarf syndrome. The results suggest that a diagnostic evaluation of the cortical olfactory response at an early age may provide indications for subsequent genetic screening.

Taken together, the six contributions which are part of this Special Issue of Brain Sciences dealing with ‘Olfaction as a Marker for Psychiatric and Neurological diseases’ should be of great interest for clinicians and neuroscientists interested on the current research directions in olfactory dysfunctions. Future studies should continue characterizing olfactory dysfunctions in psychiatric and neurological diseases and probing whether olfactory deficits in patients can apply as diagnostic and/or prognostic biomarkers for therapeutic responses in patients.

## Data Availability

Not applicable.
